# Beyond Serology: A Meta‐Analysis of Advancements in Molecular Detection of *Brucella* spp. in Seronegative Animals and Biological Samples

**DOI:** 10.1002/vms3.70200

**Published:** 2025-01-10

**Authors:** Md. Sadequl Islam, Md. Ahsan Habib, Nasrin Sultana Tonu, Md. Samiul Haque, Md. Mostafizer Rahman

**Affiliations:** ^1^ Department of Anatomy and Histology, Faculty of Veterinary and Animal Science Hajee Mohammad Danesh Science and Technology University Dinajpur Bangladesh; ^2^ Department of Animal Science and Nutrition, Faculty of Veterinary and Animal Science Hajee Mohammad Danesh Science and Technology University Dinajpur Bangladesh; ^3^ Upazila Livestock Office and Veterinary Hospital, Barura Cumilla Bangladesh; ^4^ Department of Genetics and Animal Breeding, Faculty of Veterinary and Animal Science Hajee Mohammad Danesh Science and Technology University Dinajpur Bangladesh; ^5^ Department of Microbiology, Faculty of Veterinary and Animal Science Hajee Mohammad Danesh Science and Technology University Dinajpur Bangladesh

**Keywords:** *Brucella* detection, molecular diagnostics, seronegative animals, zoonotic disease

## Abstract

**Background:**

Brucellosis is a zoonotic disease caused by *Brucella* spp., affecting various animals and humans, leading to significant economic and public health impacts. Traditional diagnostic methods, mainly serological, often fail to detect seronegative carriers, which continue to spread the infection.

**Objective:**

This review aims to highlight advancements in molecular diagnostics that address these limitations.

**Methods:**

A systematic search of PubMed, Web of Science and Scopus was conducted, focusing on studies using seronegative, PCR, qPCR and biosensor‐based techniques. Data extraction and meta‐analyses were performed, evaluating pooled detection rates and heterogeneity.

**Results:**

Through analysis of existing studies, we review key molecular techniques, including PCR, LAMP and biosensor‐based assays, which offer high sensitivity and specificity by detecting bacterial DNA directly, thus overcoming the challenges of antibody‐based tests. Meta‐analysis of detection rates across different studies showed significant variability, with rates ranging from 0.96% to 100%, highlighting differences in sample types, animal species and regions. The pooled detection proportion from random‐effects models was 35.08%, indicating that many seronegative animals still carry *Brucella* spp. A forest plot analysis further confirmed heterogeneity in detection, underlining the importance of using molecular diagnostics alongside serological tests to identify hidden carriers.

**Conclusion:**

Innovations like nanoparticle‐enhanced biosensors and CRISPR‐Cas systems show promise for rapid, on‐site diagnostics. The findings suggest that integrating molecular methods with traditional serology can improve surveillance and disease management. Future research should focus on developing portable, field‐ready diagnostic devices and standardised protocols, along with exploring novel biomarkers to detect latent infections. A collaborative One Health approach, involving veterinary, public health and environmental sectors, is essential for comprehensive disease control and eradication efforts.

## Graphical abstract

Molecular diagnostics, such as PCR and biosensor‐based techniques, offer high sensitivity in detecting *Brucella* spp. in seronegative animals, overcoming the limitations of traditional serological methods. The pooled detection rate of 35.08% suggests a substantial number of hidden carriers. Innovative methods like nanoparticle‐enhanced biosensors and CRISPR‐Cas systems show promise for rapid, on‐site diagnostics, highlighting the need for integrating molecular techniques with traditional serology for better disease control.

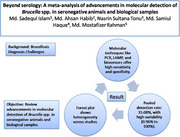

## 1 Introduction

Brucellosis is a significant zoonotic disease caused by bacteria of the genus *Brucella*, which includes 10 distinct species known for their host specificity. These species primarily infect domestic animals such as *Brucella melitensis* (goats), *Brucella abortus* (cattle), *Brucella suis* (swine) and *Brucella ovis* (sheep). Other species affect specific animals, including *Brucella canis* (dogs), *Brucella neotomae* (desert mice), *Brucella cetacea* (cetaceans), *Brucella pinnipedia* (seals), *Brucella microti* (voles) and *Brucella inopinata* (unknown host) (O'Callaghan and Whatmore 2011; Montagnaro et al. 2020). Humans contract the disease through direct contact with infected animals or their tissues, particularly reproductive organs, or by consuming contaminated animal products such as unpasteurized milk and cheese (Islam et al. 2023). With an estimated 500,000 new cases of human brucellosis reported annually worldwide (Zange and Scholz 2023), it is one of the most common zoonotic diseases, causing a range of symptoms, including fever, joint pain, fatigue, and more serious complications like arthritis and endocarditis (Islam et al. 2019; Pereira et al. 2020).

The primary transmission route to humans is occupational exposure, placing those who work closely with livestock‐such as veterinarians, farmers, and slaughterhouse workers‐at heightened risk. In many parts of the world, particularly in regions where livestock management practices and public health infrastructure are limited, brucellosis remains a serious public health issue (Islam et al. 2023). In livestock, brucellosis has significant economic impacts due to its effect on reproduction. The infection causes abortions, infertility, and reduced milk production (El‐Diasty et al. 2018). The disease is endemic in parts of Africa, the Middle East, Central Asia and Latin America, where animal brucellosis is an ongoing problem for both agriculture and public health (Rossetti, Arenas‐Gamboa, and Maurizio 2017).

Brucellosis in animals often leads to chronic infections, which are either asymptomatic or subclinical, especially in wildlife and livestock. However, when clinical signs are present, they predominantly involve reproductive losses, which lead to reduced herd sizes and decreased agricultural productivity. In addition, brucellosis can hinder the international trade of livestock and dairy products due to stringent disease control measures required by importing countries (Godfroid 2017). The control of brucellosis depends largely on early detection and vaccination. However, there is no effective vaccine for human use, and animal vaccination programmes, where they exist are often inconsistent or inadequately implemented (Huang et al. 2024).

The diagnosis of brucellosis in animals traditionally relies on serological techniques, including the Rose Bengal Plate Test (RBPT), complement fixation tests (CFTs) and enzyme‐linked immunosorbent assays (ELISA) (Legesse et al. 2023). These methods detect antibodies produced in response to *Brucella* infection and have been widely used in surveillance and control programmes. However, these tests have significant limitations, particularly in detecting chronic infections, as antibody levels decline over time. During the chronic phase, antibody titres may drop to levels too low to be detected by serological methods (Islam et al. 2018). Moreover, *Brucella* bacteria can evade the host immune system by residing inside host cells, further complicating diagnosis as infected animals may not produce sufficient antibodies to trigger positive results in serological tests (Milgroom 2023).

This limitation in serological testing becomes particularly problematic when dealing with seronegative carriers—animals that do not exhibit detectable antibody levels yet are still infected with *Brucella*. These animals can continue to harbour the bacteria and spread the infection to other animals or humans, posing a serious challenge to control programmes (Freire et al. 2024). The presence of such seronegative carriers in livestock herds allows the disease to persist within populations, perpetuating the cycle of infection.

A study conducted in the Sistan–Baluchistan province of Iran illustrates the challenges posed by seronegative carriers. The study aimed to detect *Brucella* in blood and lymph node samples collected from slaughtered male camels. While traditional serological methods like RBPT and the serum agglutination test (SAT) were used, a significant portion of infected animals were not detected. Out of 2854 camel blood samples, only a small percentage tested positive with serological methods. However, PCR testing on lymph node samples from seronegative animals revealed that 6.6% of these animals were infected with *Brucella* (Dadar and Alamian 2020). This finding highlights the inadequacy of relying solely on serological tests for brucellosis diagnosis, particularly when dealing with seronegative carriers. A similar issue has been observed in bovine brucellosis. Some studies indicate that seronegative animals can still be infected with *Brucella* spp., as PCR analysis has detected *Brucella* DNA in animals that tested negative on serological tests. These seronegative animals may be in the early stages of infection or may have a low bacterial load, which makes them undetectable by traditional serological methods (Batrinou et al. 2022). This reinforces the need for molecular diagnostic methods, such as polymerase chain reaction (PCR), to complement serological testing in identifying infected animals and preventing further spread of the disease.

Molecular diagnostic methods, particularly PCR, have become increasingly important in the detection of *Brucella* spp. PCR is a highly sensitive technique that can detect bacterial DNA in various animal samples, including blood, milk and tissues (Legesse et al. 2023). Unlike serological tests, PCR does not depend on the host's immune response but directly identifies *Brucella* DNA, making it a valuable tool for detecting infections in seronegative animals. PCR assays targeting the *IS711* gene, which is unique to the *Brucella* genus, have proven particularly effective in identifying infections where serological tests fail (Ntivuguruzwa et al. 2022). One study involving wild boars demonstrated that *IS711*‐based PCR could detect Brucella DNA in animals where bacterial isolation methods had failed and serological tests yielded negative results (Hinić et al. 2009). One major advantage of molecular methods like PCR is their versatility in detecting *Brucella* in various sample types, such as blood, milk, tissues and semen, making them indispensable for use in endemic regions. Given the limitations of serological tests, integrating molecular diagnostics into routine surveillance protocols is crucial for improving detection rates, particularly in regions where brucellosis remains endemic (Loubet et al. 2024).

Recent advancements in brucellosis diagnosis have focused on more sensitive and rapid diagnostic technologies. Magnetic nanoparticle (MNP)‐based DNA biosensors are one such promising tool. These biosensors, when combined with frequency mixing magnetic detection (FMMD), enable the selective detection of *Brucella* DNA by hybridising with target DNA sequences. This method allows for the rapid detection of low levels of bacterial DNA, with a detection time of approximately 10 min, even in field settings. Such technologies show promise for point‐of‐care (POC) diagnostics (Abuawad et al. 2023). Another innovative approach involves immuno‐surface plasmon resonance (SPR) biosensors, which provide a portable, low‐cost detection method with a detection limit as low as 2.8 bacteria/mL, without the need for DNA amplification or complex laboratory equipment (Pasquardini, Cennamo et al. 2023a).

This review addresses the critical gap in brucellosis diagnostics by emphasising the need for molecular detection of *Brucella* spp. in seronegative animals. Traditional serological methods, although widely used, often miss infections in animals without detectable antibodies, leading to underreporting and continued transmission. By reviewing various studies and pooling data through meta‐analysis, this review provides compelling evidence in favour of molecular techniques, particularly PCR‐based methods, to detect *Brucella* in seronegative animals. This shift is especially crucial in regions where the disease is endemic, and the reliance on serological methods alone has contributed to the persistence of the disease. The findings of this review also underline the broader economic and public health benefits of integrating molecular diagnostics into regular surveillance and disease control programmes.

## 2 Materials and Methods

This review aimed to assess advancements in molecular detection methods for *Brucella* spp. in animals and biological samples, with a focus on seronegative animals. A systematic approach was applied, focusing on studies from diverse regions and covering various molecular techniques such as PCR, quantitative PCR (qPCR), loop‐mediated isothermal amplification (LAMP), and biosensor‐based assays. A comprehensive literature search was performed using scientific databases such as PubMed, Web of Science and Scopus, with the search completed in August 2024. Keywords such as ‘*Brucella* detection’, ‘seronegative animals’, ‘molecular diagnostics’ and ‘PCR’ were utilised. Studies included in the review were limited to those focusing on seronegative animal cases, where molecular diagnostics were employed to detect *Brucella* spp. Only articles in English were included. Data extraction was carried out by two independent reviewers, who identified key characteristics from each study, including the sample type (blood, milk, tissues, etc.), detection method, geographical region, and the reported detection rate of *Brucella* in seronegative animals. Any discrepancies in data extraction were resolved through consensus or by consulting a third reviewer.

Meta‐analyses were performed to assess pooled detection rates across different sample types and regions using random‐effects models, accounting for variability across studies. Forest plots were generated to visualise the heterogeneity in detection rates. Heterogeneity was assessed using *I* ^2^ statistics, and publication bias was evaluated with Egger's and Begg's tests.

The review analysed various molecular techniques, with PCR, LAMP and qPCR being the most frequently used. Studies using advanced biosensor‐based detection methods, such as nanoparticle‐enhanced assays and CRISPR‐Cas systems, were also included. Each diagnostic technique was evaluated based on sensitivity, specificity and ability to detect *Brucella* spp. in seronegative animals. The statistical analyses were performed using MedCalc statistical software version 23.0.5 to ensure the accuracy of the information presented. Pooled detection proportions were calculated, and 95% confidence intervals (CIs) were reported for each study. Since this is a review study based on previously published data, ethical approval was not required. All data used were from publicly available studies.

## 3 Tradition *Brucella* Detection Methods

Serological methods for detecting *Brucella* spp. in animals and animal products include a variety of tests, each with its own advantages and limitations. Some of the common tests used for *Brucella* detection are the standard tube agglutination test (STAT), RBPT, 2‐mercaptoethanol test (MET), rivanol test, milk ring test (MRT), CFT, radioimmunoassay (RIA), indirect ELISA (iELISA), competitive enzyme immunoassay (cELISA) and fluorescence polarisation assay (FPA). While these tests are widely used, not all of them are ideal for every scenario, and they come with various challenges. STAT is a commonly used serological test to detect antibodies in animals, but it suffers from several drawbacks. The test often produces cross‐reactivity with antibodies from other bacteria, such as *Yersinia enterocolitica* or *Escherichia coli*, leading to false‐positive results (Islam et al. 2018). In addition, it is not very sensitive during the early stages of infection or in chronic cases, which can result in false negatives. STAT is also unable to distinguish between antibodies generated by natural infection and those produced due to vaccination. RBPT is a rapid screening test used to detect *Brucella* antibodies. While it is easy to use and cost‐effective, its main limitation is its relatively low specificity. The test can cross‐react with antibodies produced by infections with other bacteria, such as *Francisella tularensis* and *Salmonella* spp., resulting in false positives. In addition, the RBPT can yield false negatives, particularly in animals with low antibody titres or in the early stages of infection (Legesse et al. 2023).The MET is used to differentiate between IgM and IgG antibodies, with the former indicating recent infection. While this test can help identify active infections, it is complex to perform and interpret. False positives can occur due to non‐specific antibody binding, and false negatives can happen in chronically infected animals where IgM levels have declined. Like other agglutination‐based tests, the MET cannot distinguish between infection‐induced antibodies and vaccine‐induced ones (Freire et al. 2024).The rivanol test is designed to reduce non‐specific reactions by precipitating out non‐*Brucella*‐specific antibodies. While this increases specificity, the test is labour‐intensive and time‐consuming, making it less practical for large‐scale testing. In addition, rivanol can affect antibody–antigen reactions, leading to occasional false negatives. Like many other serological tests, it also cannot differentiate between antibodies from infection and vaccination (Delano, Mischler, and Underwood 2002).The MRT is a screening test for detecting *Brucella* antibodies in milk. While it is useful for monitoring dairy herds, the test is less reliable in individual animals, particularly when milk composition or fat content varies significantly. False positives can occur due to non‐specific binding in high‐fat milk or poor sample handling, and false negatives can arise in animals with low antibody titres (Islam et al. 2023). In addition, MRT is not suitable for detecting *Brucella* in milk products such as cheese or yoghurt. CFT is highly specific and often used as a confirmatory test. However, it requires a well‐maintained laboratory setup and is technically demanding. In addition, CFT is prone to interference from factors like poor sample quality and the presence of complement‐inhibiting substances, which can lead to false negatives. Like other serological tests, CFT also struggles with distinguishing between antibodies from natural infection and those from vaccination (Legesse et al. 2023). RIA is a sensitive test that uses radioactively labelled antigens to detect antibodies against *Brucella*. Despite its high sensitivity, the use of radioactive materials makes it expensive and difficult to implement, especially in resource‐limited settings. The handling and disposal of radioactive waste require specialised facilities and trained personnel, limiting its practical application. Furthermore, the test's high sensitivity may result in false positives, especially when cross‐reacting antibodies are present (World Health Organization [WHO] 2006). iELISA is widely used for detecting *Brucella* antibodies due to its high sensitivity and ability to process large numbers of samples simultaneously. However, iELISA can also suffer from cross‐reactivity with other bacteria, leading to false positives. While it is more sensitive than agglutination tests, it may still produce false negatives in animals with low antibody levels or in early infection. The iELISA is also unable to differentiate between vaccinated and naturally infected animals (Godfroidet al. 2013). cELISA is a modification of iELISA that improves specificity by reducing cross‐reactivity. It is particularly useful in distinguishing between antibodies from vaccinated and naturally infected animals. Despite these advantages, cELISA is technically more complex and requires specialised reagents, which can be costly and difficult to obtain in certain regions. In addition, it can still yield false negatives in cases of low antibody titres or chronic infection (WHO 2006). FPA is a sensitive and specific test that measures the rotation of fluorescent‐labelled antigens in the presence of antibodies (Montagnaro et al. 2008). While FPA has high specificity and is less prone to cross‐reactivity than some other tests, it requires specialised equipment and technical expertise, limiting its availability in certain regions. Like other serological tests, FPA cannot distinguish between antibodies from infection and vaccination, which can complicate the interpretation of the results (Suo et al. 2021). Serological methods for *Brucella* detection have several limitations. They detect antibodies but cannot distinguish between active and past infections, leading to potential misinterpretation, especially in animals that have cleared the infection but still test positive. This can result in unnecessary culling or control measures, even though the animals are no longer infectious. In addition, immunosuppression in animals due to factors like stress, malnutrition or disease can weaken the immune response, causing false‐negative results in serological tests. This is a significant concern in livestock under intensive farming conditions, where overcrowding and poor nutrition compromise immune function. Moreover, serological methods are not suitable for detecting *Brucella* in animal products like milk, cheese or meat. Tests such as the MRT can identify antibodies in milk; however, they are less dependable than blood tests and may be influenced by factors such as the fat content in milk or incorrect handling. As a result, they are not adequate for detecting contamination in animal products(WHO 2006). The sensitivity and specificity of serological tests for *Brucella* detection can vary significantly between animal species. For example, the Rose Bengal test (RBT) is highly sensitive in cattle but less reliable in small ruminants like sheep and goats. Similarly, the CFT, commonly used in cattle, may have lower specificity in wildlife and certain small ruminants. The SAT, while effective in cattle, can produce false positives due to cross‐reactivity with other bacteria, particularly in goats and sheep. ELISA typically shows high sensitivity and specificity in cattle but may have reduced accuracy in wildlife and non‐domestic species. This variability necessitates species‐specific considerations for diagnosing brucellosis, as tests designed for one species may yield inconsistent results in others. In wildlife, which can serve as reservoirs for *Brucella*, developing specific serological tests is challenging due to species diversity and difficulty in obtaining large sample sizes for validation. In addition, the antibody response to *Brucella* infection can vary depending on the animal species, the *Brucella* strain, and the disease stage. For instance, cattle infected with *B. abortus* may develop a strong immune response, while goats infected with *B. melitensis* may show more variable antibody levels. Furthermore, serological tests may be more suited to detecting certain antibodies (e.g., IgM in acute infections or IgG in chronic cases), leading to inconsistent results based on the infection stage (WHO 2006).

## 4 Key Findings

### 4.1 Variability and Heterogeneity in *Brucella* Detection Rates: A Meta‐Analysis of Seronegative Animal

Table 1 highlights significant variability in the detection rates of *Brucella* spp. from seronegative animals across different animal species, sample types and geographical regions using methods like PCR, qPCR and biochemical tests. High detection rates were observed in certain species, such as camels in Sudan (100% in blood samples), pigs in Kenya (100% in blood samples) and dogs in Brazil (100% in blood samples), indicating a high positivity for *Brucella* in these animals. In contrast, cow milk samples generally showed lower detection rates, with the highest detection rate in Tajikistan (8.3%) and the lowest in Iran (2.09%). Species‐specific differences were also noted, with rams (semen, 66.66%), bulls (semen, 68%), and cows (liver, spleen and lymph nodes, 76.6%) exhibiting relatively high detection rates, especially in reproductive organs and tissues. The sample type significantly impacted detection rates, as uterine discharge in goats (40%) and cows (27.27%) yielded higher positivity compared to vaginal exudates and milk, which had much lower rates (0.18% in goats, 0.96% in cows).

**TABLE 1:** Molecular and biochemical detection rates of *Brucella* spp. in seronegative animals and biological samples across various regions.

Species	Type of sample	Country/region	Detection method	Seronegative sample size	Number of positive sample by PCR/culture/biochemical test	Detection rate (%)	References
Cow	Milk	South Taranaki (New Zealand)	Biochemical	446	22	4.93	Rickard (1965)
Cow	Milk, vaginal mucus	New South Wales, Australia	Biochemical	944	42	4.4	Mylrea (1972)
Ram	Semen	Eastern Anglia	Biochemical	9	6	66.66	Bulgin (1990)
Cow	Milk	Iran	Biochemical	5686	119	2.09	Zowghi, Ebadi, and Mohseni (1990)
Sheep	Blood, lymphoid tissue	Turkey	PCR	234	6	2.56	Ilhan et al. (2008)
Wild boars	Tissues from reproductive organs, lungs and spleen	Switzerland	PCR	144	16	11.1	Hinić et al. (2009)
Camel	Blood	Sudan	qPCR	118	118	100	Gwida et al. (2011)
Goat	Vaginal exudate	Mexico	PCR	18	1	0.18	Herrera et al. (2011)
Bull	Semen	Brazil	PCR	88	27	30.68%	Junqueira Junior et al. (2012)
Cow	Vaginal exudate	Pichucalco, Chiapas (Mexico)	PCR	209	2	0.96	Arellano‐Reynoso et al. (2013)
Bull	Semen	Brazil	PCR	100	68	68	Lourencetti et al. (2017)
Cow	Milk	Tajikistan	qPCR	552	46	8.3	Lindahl‐Rajala et al. (2017)
Cow	Placenta, stomach content from aborted foetus	Egypt	PCR	21	4	19.04	Wareth et al. (2016)
Cow	Milk	Bangladesh	PCR	342	6	1.75	Islam et al. (2018)
Cow	Milk	Batna, Algeria	PCR	65	2	3.08	Sabrina et al. (2018)
Cow	Uterine discharge	Egypt	PCR	11	3	27.27	El‐Diasty et al. (2018)
Goat	Uterine discharge	Egypt	PCR	5	2	40	El‐Diasty et al. (2018)
Cow	Liver, spleen, lymphnode	South Africa	PCR	25	11	44	Kolo et al. (2019)
Pig	Blood	Kenya	qPCR	2	2	100	Akoko et al. (2020)
Camel	Lymphnodes	Iran	PCR	30	2	6.6	Dadar and Alamian (2020)
Cow	Vaginal swab, milk	Rwanda	PCR	27	4	14.81	Ntivuguruzwa et al. (2022)
Cow	Blood, milk	Greece	PCR	2	2	100	Batrinou et al. (2022)
Cow	Milk	Iran and Tehran	PCR	2	2	100	Abnaroodheleh et al. (2023)
Dog	Blood	Brazil	PCR	250	250	100	Schiavo et al. (2024)
Cow	Spleen, liver, lymphnode	South Africa	PCR	41	31	76.6	Mazwi et al. (2024)
Sheep	Spleen, liver, lymphnode	South Africa	PCR	15	13	86.7	Mazwi et al. (2024)
Pig	Spleen, liver, lymphnode	South Africa	PCR	2	2	100	Mazwi et al. (2024)

**FIGURE 1:**
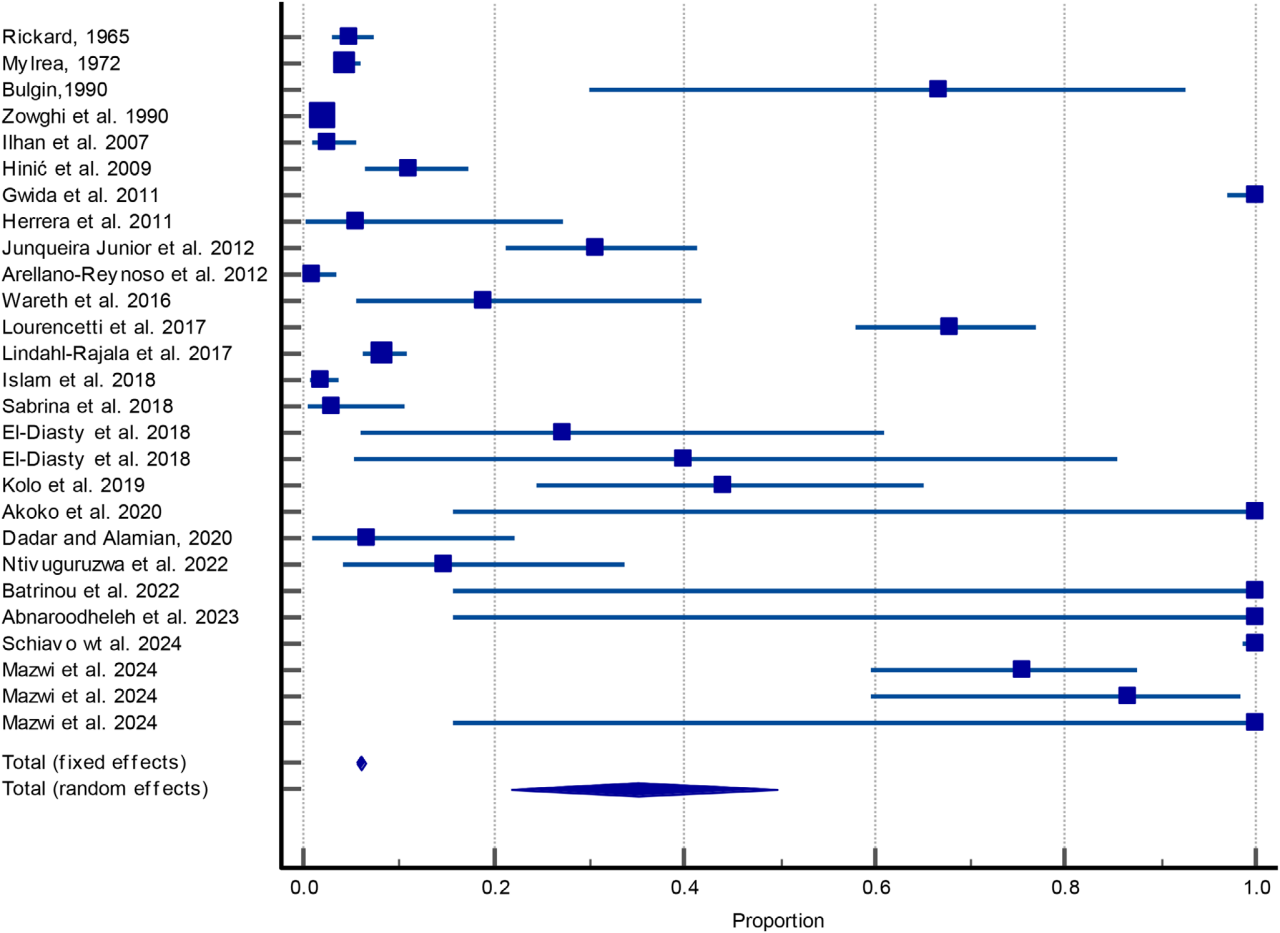
Forest plot of detection rates of *Brucella* spp. across different studies and sample types. The plot displays proportions of seronegative animals testing positive for *Brucella* spp. using various detection methods (PCR, qPCR, biochemical tests) across multiple studies from different countries. The horizontal lines represent the 95% confidence intervals for the detection rate in each study, and the squares indicate the point estimates. The size of the squares reflects the weight of each study in the meta‐analysis, while the diamond at the bottom represents the overall effect estimate (total random effects).

The forest plot (Figure 1) illustrates the detection rates of *Brucella* spp. across various studies, animal species and detection methods. The individual study proportions vary widely, ranging from as low as approximately 0.02 (Herrera et al. 2011) to 1.0 (Gwida et al. 2011; Schiavo et al. 2024; Akoko et al. 2020), indicating significant heterogeneity in *Brucella* detection rates. Several studies, such as Lourencetti et al. (2017) and Mazwi et al. (2024), report detection rates approaching 0.7 or higher, suggesting high positivity in their respective sample populations. The CIs of some studies, such as Junqueira Junior et al. (2012) and Kolo et al. (2019), are relatively wide, indicating variability or smaller sample sizes, while other studies, like those by Gwida et al. (2011) and Schiavo et al. (2024), display narrower CIs, reflecting higher precision in detection estimates. The overall effect, represented by the diamond at the bottom, suggests a pooled detection proportion that lies around 0.2, indicating a significant proportion of seronegative animals tested positive for *Brucella* spp. using advanced molecular and bacterial detection methods. The broad range of detection rates across studies highlights the importance of study design, animal species, sample types and geographic region in influencing *Brucella* detection outcomes.

The meta‐analysis of *Brucella* detection rates in seronegative animals and biological samples shows a wide range of detection proportions across different studies, with individual rates ranging from as low as 0.957% (Arellano‐Reynoso et al. 2013) to 100% (Gwida et al. 2011; Schiavo et al. 2024). In this meta‐analysis, data from 27 studies were pooled to assess the proportion of positive cases detected by various methods (PCR, biochemical tests) in a wide range of sample sizes. The total sample size across all studies was 9388, with an overall fixed‐effect estimate of the proportion of positive cases being 6.03% (95% CI: 5.56%–6.53%). However, when using a random‐effects model, the overall proportion increased to 35.08% (95% CI: 21.77%–49.68%) (Table 2).

**TABLE 2:** Meta‐analysis proportion for the detection rates of *Brucella* spp. in seronegative animals and biological samples through molecular or biochemical test.

Study	Sample size	Proportion (%)	95% CI	Weight (%)	
				Fixed	Random
Rickard (1965)	446	4.933	3.117–7.373	4.75	4.08
Mylrea (1972)	944	4.449	3.225–5.967	10.04	4.09
Bulgin (1990)	9	66.667	29.930–92.515	0.11	3.48
Zowghi, Ebadi, and Mohseni (1990)	5686	2.093	1.737–2.499	60.40	4.10
Ilhan et al. (2008)	234	2.564	0.947–5.497	2.50	4.07
Hinić et al. (2009)	144	11.111	6.486–17.416	1.54	4.05
Gwida et al. (2011)	118	100.000	96.922–100.000	1.26	4.04
Herrera et al. (2011)	18	5.556	0.141–27.294	0.20	3.75
Junqueira Junior et al. (2012)	88	30.682	21.288–41.420	0.95	4.02
Arellano‐Reynoso et al. (2013)	209	0.957	0.116–3.414	2.23	4.07
Wareth et al. (2016)	21	19.048	5.446–41.907	0.23	3.79
Lourencetti et al. (2017)	100	68.000	57.923–76.978	1.07	4.03
Lindahl‐Rajala et al. (2017)	552	8.333	6.165–10.959	5.87	4.09
Islam et al. (2018)	342	1.754	0.646–3.779	3.64	4.08
Sabrina et al. (2018)	65	3.077	0.375–10.677	0.70	3.99
El‐Diasty et al. (2018)	11	27.273	6.022–60.974	0.13	3.57
El‐Diasty et al. (2018)	5	40.000	5.274–85.337	0.064	3.16
Kolo et al. (2019)	25	44.000	24.402–65.072	0.28	3.84
Akoko et al. (2020)	2	100.000	15.811–100.000	0.032	2.57
Dadar and Alamian (2020)	30	6.667	0.818–22.074	0.33	3.88
Ntivuguruzwa et al. (2022)	27	14.815	4.189–33.731	0.30	3.85
Batrinou et al. (2022)	2	100.000	15.811–100.000	0.032	2.57
Abnaroodheleh et al. (2023)	2	100.000	15.811–100.000	0.032	2.57
Schiavo et al. (2024)	250	100.000	98.535–100.000	2.67	4.07
Mazwi et al. (2024)	41	75.610	59.695–87.637	0.45	3.93
Mazwi et al. (2024)	15	86.667	59.540–98.342	0.17	3.69
Mazwi et al. (2024)	2	100.000	15.811–100.000	0.032	2.57
Total (fixed effects)	9388	6.033	5.560–6.533	100.00	100.00
Total (random effects)	9388	35.075	21.771–49.684	100.00	100.00

The studies included varied widely in their sample sizes and proportions, as seen in studies like Zowghi, Ebadi, and Mohseni (1990) with a large sample size of 5686 and a lower detection rate of 2.09%, compared to smaller studies like Schiavo et al. (2024) with 250 cases and a 100% detection rate. This heterogeneity is further supported by the heterogeneity test (*Q* = 3266.20, *p* < 0.0001), with an *I* ^2^ value of 99.20%, indicating very high variability between the studies that is not due to chance (Table 3). The observed heterogeneity in the meta‐analysis of *Brucella* spp. detection in seronegative animals underscores the need for a more in‐depth exploration of the factors contributing to this variability. One significant source of heterogeneity is sample handling and processing. Variations in how biological samples are collected, stored and processed across studies can influence detection rates, particularly when bacterial degradation occurs due to suboptimal storage conditions or delayed processing. Standardising sample handling protocols, such as maintaining consistent refrigeration or freezing times, would help mitigate this issue (Millwood and Walters 2020). In addition, regional variations in livestock management practices, environmental factors, and the prevalence of *Brucella* species may account for discrepancies in detection outcomes. Studies conducted in regions with stricter biosecurity measures tend to report lower detection rates compared to areas with less rigorous management, and endemic regions may see more subclinical cases, further complicating diagnostics (Mwatondo et al. 2023). Furthermore, differences in diagnostic methods contribute significantly to the heterogeneity observed. Molecular techniques, such as PCR and qPCR, offer greater sensitivity in detecting *Brucella* in seronegative animals, while traditional serological methods often fail to identify infections in latent or chronic stages. Variability in molecular assay design, such as the choice of specific molecular markers, may also lead to inconsistent detection across studies (Scholzet al. 2023). Species‐specific differences also play a role, as detection rates vary widely among different animal species, with some, such as camels and pigs, showing higher rates of positivity compared to cows or goats. These variations suggest that diagnostic approaches may need to be tailored to the specific biology of each species. Moreover, the type of biological sample used for testing, such as reproductive tissues versus milk or blood, significantly impacts detection success, with tissues from reproductive organs generally yielding higher detection rates (Mengele et al. 2023). Addressing these factors through standardisation of protocols, regional adjustments and species‐specific strategies could reduce variability and enhance the consistency of *Brucella* detection across studies, ultimately strengthening the reliability of brucellosis surveillance and control efforts.

**TABLE 3:** Test for heterogeneity for the detection rates of *Brucella* spp.

*Q*	3266.2005
DF	26
Significance level	*p* < 0.0001
*I* ^2^ (inconsistency)	99.20%
95% CI for *I* ^2^	99.09–99.30

Egger's test for publication bias showed a significant intercept (6.99, 95% CI: 1.93–12.06, *p* = 0.0088), indicating potential bias in smaller studies. In contrast, Begg's test was not significant (Kendall's tau = 0.2464, *p* = 0.0714), suggesting no strong evidence of publication bias (Table 4). While both Begg's and Egger's tests assess publication bias, they differ in sensitivity. Begg's test, based on rank correlation, is less sensitive, particularly in smaller studies, so a non‐significant result does not completely rule out bias. Egger's test, using regression analysis, is more sensitive and better at detecting subtle biases, especially in small studies. The significant result from Egger's test suggests potential bias, despite the non‐significant Begg's test, likely due to Egger's higher sensitivity. Thus, Egger's test identified bias that Begg's test might have missed.

**TABLE 4:** Publication bias test for the detection rates of *Brucella* spp.

Egger's test	
Intercept	6.9904
95% CI	1.9250–12.0558
Significance level	*p* = 0.0088
Begg's test	
Kendall's tau	0.2464
Significance level	*p* = 0.0714

### 4.2 Diagnostic Challenges in Low‐Resource Settings

In low‐resource settings, traditional serological methods frequently fail to detect *Brucella* in seronegative animals, leaving latent infections undiagnosed. Molecular diagnostics such as PCR, real‐time PCR (qPCR) and ITS‐PCR have emerged as critical tools to uncover these hidden infections. For instance, in Egypt, El‐Diasty et al. (2018) found that *B. melitensis* and *B. abortus* were present in uterine discharge samples from cows that had tested seronegative, proving that serological methods alone are insufficient. Similarly, Dadar and Alamian (2020) showed that 6.6% of lymph node samples from seronegative camels in Iran tested positive for *Brucella* via PCR.

Studies in Bangladesh (Islam et al. 2018) revealed that 1.75% of seronegative cow milk samples were positive for *Brucella* DNA, and in Tajikistan, Lindahl‐Rajala et al. (2017) found 8.3% positivity in cow milk samples using qPCR. These examples highlight the importance of molecular diagnostics in low‐resource settings where traditional serology often underestimates infection rates. In regions like South Africa, Kolo et al. (2019) used PCR to detect *Brucella* in 44% of cow samples from the spleen, liver and lymph nodes that were seronegative by traditional methods.

Molecular techniques like qPCR have also proven their utility in other resource‐limited regions. For example, Gwida et al. (2011) found a 100% detection rate in seronegative camel blood samples from Sudan using qPCR, emphasising the technique's critical role in such environments. This illustrates the necessity of implementing advanced molecular diagnostics in low‐resource settings to improve detection accuracy and prevent the silent spread of brucellosis among livestock.

### 4.3 Wildlife as Reservoirs vs. Livestock Transmission

Wildlife can act as reservoirs for *Brucella*, complicating efforts to control its spread in livestock. A study by Hinić et al. (2009) in Switzerland detected *Brucella* DNA in 11.1% of wild boar tissue samples, suggesting that wildlife can harbour the pathogen without obvious symptoms. However, while this demonstrates wildlife as a potential reservoir, evidence of direct spillover from wildlife to livestock remains limited. For example, Mazwi et al. (2024) found *Brucella* in 86.7% of spleen, liver and lymph node samples from sheep and 75.6% of samples from cows in South Africa, but these animals were seronegative, indicating subclinical infection. This study focuses on livestock infection but does not provide direct evidence of transmission from wildlife, making it insufficient for demonstrating spillover dynamics. In North America, Herrera et al. (2011) detected *Brucella* DNA in 0.18% of vaginal exudate samples from seronegative goats in Mexico, further highlighting the challenge of detecting undiagnosed infections in livestock. Lourencetti et al. (2017) in Brazil similarly found that 68% of semen samples from seronegative bulls were positive for *Brucella*, revealing the risk of silent infection in apparently healthy livestock. These studies emphasize the need for more robust livestock surveillance to identify undetected carriers, though they do not address the potential for wildlife‐to‐livestock transmission.

While *Brucella* has been detected in both wildlife and livestock, the connection between wildlife reservoirs and livestock transmission remains unclear, particularly regarding spillover. Wildlife–livestock interactions could theoretically increase the risk of cross‐species transmission in regions where both populations overlap, but direct evidence for this remains limited. For example, in South Africa, Mazwi et al. (2024) found *Brucella* in 100% of pig tissue samples. However, they did not investigate or report interactions between the sampled pigs and wildlife, leaving the role of wildlife in transmission uncertain. Further studies explicitly focusing on direct wildlife‐to‐livestock transmission are needed to understand these dynamics. Without this evidence, wildlife surveillance cannot yet be effectively integrated into livestock disease management strategies. Comprehensive monitoring that includes both wildlife and livestock will be critical to preventing the spread of *Brucella*, but current data do not support a definitive wildlife‐to‐livestock transmission model.

### 4.4 Regional Variability in *Brucella* Detection

The detection rates of *Brucella* vary significantly between regions, largely due to differences in diagnostic capabilities, livestock management practices and pathogen prevalence. Studies across the globe have demonstrated this variability. For example, in Turkey, Ilhan et al. (2008) found that 2.56% of blood and lymphoid tissue samples from seronegative sheep tested positive for *Brucella* using PCR, while Sabrina et al. (2018) reported a 3.08% detection rate in cow milk samples from Algeria using the same method.

A significant variation is also observed in Central and South America. Arellano‐Reynoso et al. (2013) detected *Brucella* DNA in 0.96% of vaginal exudate samples from seronegative cows in Mexico, highlighting low but consistent infection rates. Meanwhile, Junqueira Junior et al. (2012) found *Brucella* in 30.68% of semen samples from seronegative bulls in Brazil, indicating the presence of the disease in reproductive tissues that might otherwise go unnoticed. In Iran, Zowghi, Ebadi, and Mohseni (1990) showed a low detection rate of 2.09% in milk samples from seronegative cows, while Abnaroodheleh et al. (2023) reported 100% positivity in the same country for a small sample size of two cases. These findings emphasize how molecular diagnostics, such as PCR, provide more accurate detection, even in regions with lower overall infection rates.

The studies from Kenya (Akoko et al. 2020) detected *Brucella* in 100% of seronegative pig blood samples using qPCR, revealing a high burden of the disease in this species. Similarly, Schiavo et al. (2024) reported a 100% detection rate in seronegative dog blood samples in Brazil, further showcasing the variability in *Brucella* prevalence based on species and geography. The findings from these diverse studies emphasize the need for region‐specific diagnostic protocols that incorporate molecular methods like PCR to improve detection rates. Tailoring diagnostic approaches based on local epidemiological data is essential to identify silent carriers of *Brucella*, particularly in regions where traditional serological methods are inadequate. In such areas, molecular techniques are critical for accurate detection, ensuring that *Brucella* transmission is effectively prevented.

## 5 Molecular and Biosensor‐Based Detection Techniques for *Brucella* spp

Molecular detection techniques for *Brucella* spp. have evolved significantly over the years, providing more accurate, rapid and sensitive methods for diagnosing brucellosis in various animal species and their biological samples. These techniques are crucial for effective disease management and control, especially in regions where brucellosis is endemic. Advanced molecular techniques enable rapid, accurate, and sensitive detection, even in seronegative or subclinical cases.

PCR is one of the most widely used molecular techniques for detecting *Brucella* spp. PCR amplifies specific DNA sequences, making it possible to detect even small amounts of bacterial DNA in samples. Various PCR methods, including conventional PCR, qPCR and multiplex PCR, have been developed to identify *Brucella* spp. in different biological samples such as milk, tissues, blood and discharge. Conventional PCR is often used for its simplicity and cost‐effectiveness, while qPCR offers the advantage of quantifying the bacterial load in samples, providing both diagnostic and epidemiological insights (Islam et al. 2023). qPCR has become a gold standard for the rapid and sensitive detection of *Brucella* spp. This technique uses fluorescent dyes or probes to monitor the amplification process in real time, allowing for the quantification of bacterial DNA. qPCR is highly specific and can differentiate between various *Brucella* species, making it an invaluable tool for both clinical diagnostics and research. For instance, studies have shown that qPCR can detect *Brucella* DNA in milk samples from infected cows, providing a non‐invasive method for monitoring dairy herds (Pelerito et al. 2021). PCR and qPCR both techniques are both widely recognised for their ability to detect low levels of *Brucella* DNA, even in cases where the animal has not yet developed detectable antibodies. However, PCR may be influenced by factors such as sample quality, contamination and the presence of inhibitors, potentially leading to false negatives.

Multiplex PCR is another advanced technique that allows the simultaneous detection of multiple *Brucella* species in a single reaction. This method uses multiple sets of primers, each specific to a different *Brucella* species, enabling the identification of co‐infections and providing a comprehensive overview of the infection status in a herd. Multiplex PCR has been successfully used to detect *Brucella* spp. in various tissues and fluids, including lymph nodes, spleen and foetal tissues, highlighting its versatility and efficiency (Katsiolis et al. 2022).

Digital PCR (dPCR) is an emerging molecular technique that offers high sensitivity and precision for the detection of *Brucella* spp. dPCR partitions the DNA sample into thousands of individual reactions, allowing for the absolute quantification of target DNA molecules. This technique is particularly useful for detecting low levels of *Brucella* DNA in complex samples, such as blood or tissue biopsies. dPCR has been used to monitor *Brucella* infections in wildlife populations, providing valuable data for disease surveillance and management (Kurmanov et al. 2022).

LAMP is a relatively new molecular technique that offers several advantages over traditional PCR methods. LAMP amplifies DNA at a constant temperature, eliminating the need for thermal cycling and making the process faster and more cost‐effective. This technique is highly sensitive and specific, capable of detecting low levels of *Brucella* DNA in various samples. LAMP has been used to detect *Brucella* spp. in milk, blood and tissue samples, providing a rapid diagnostic tool that can be used in field settings where laboratory facilities are limited (Ratushna et al. 2006).

Whole‐genome sequencing (WGS) represents the gold standard for molecular typing and phylogenetic analysis of *Brucella* spp. Pelerito et al. (2021) discussed how WGS allows for the detailed characterisation of *Brucella* strains, including the identification of virulence factors, antibiotic resistance genes and phylogenetic relationships. This method has been instrumental in tracing outbreaks and understanding transmission pathways between wildlife and livestock. WGS can replace traditional Multi‐Locus Variable Number Tandem Repeat Analysis (MLVA) by providing comprehensive genetic data. For example, WGS has been used to profile strains from various geographical regions and differentiate between closely related species like *B. melitensis*, *B. abortus* and *B. suis*.

Fluorescent in situ hybridisation (FISH) is a molecular technique that uses fluorescent probes to detect‐specific DNA sequences within cells. FISH has been used to identify *Brucella* spp. in tissue samples, providing a visual confirmation of the presence of the bacteria in seronegative animals. This technique is particularly useful for studying the localization and distribution of *Brucella* within host tissues, offering insights into the pathogenesis of the infection. FISH can be combined with other molecular techniques, such as PCR, to enhance the sensitivity and specificity of *Brucella* detection (Kurmanov et al. 2022).

Next‐generation sequencing (NGS) technologies have revolutionised the field of molecular diagnostics, providing high‐throughput and detailed analysis of *Brucella* genomes. NGS allows for the simultaneous sequencing of multiple samples, providing a comprehensive overview of the genetic diversity and population structure of *Brucella* spp. This technique has been used to identify novel genetic markers and virulence factors, enhancing our understanding of *Brucella* pathogenesis and epidemiology. NGS can also be used to monitor the emergence of antibiotic‐resistant strains, providing valuable information for the development of effective treatment strategies (Pelerito et al. 2021).

Microarray analysis is another advanced molecular technique used for the detection and characterisation of *Brucella* spp. Microarrays consist of a grid of DNA probes that can hybridise with specific *Brucella* DNA sequences, allowing for the simultaneous detection of multiple genetic targets. This technique has been used to identify genetic variations and mutations associated with *Brucella* virulence and antibiotic resistance. Microarray analysis provides a high‐throughput and cost‐effective method for the comprehensive analysis of *Brucella* genomes, making it a valuable tool for both research and diagnostics. This method can detect low bacterial loads in seronegative animals (Ratushna et al. 2006).

Recent advancements in biosensor‐based and molecular techniques have significantly enhanced the detection of *Brucella* spp., offering more sensitive, rapid, and specific diagnostic tools compared to traditional methods. Various biosensor technologies have been developed to facilitate the rapid and accurate detection of *Brucella* spp. These methods are capable of detecting low bacterial loads. Yet, they remain in the early stages of development, and more validation is needed before widespread implementation.

Lateral flow assays (LFA) have been enhanced using nanoparticles to increase their sensitivity and specificity. Zhang et al. (2023) described a rapid vertical flow technique (RVFT) that utilises Prussian blue nanoparticles for the detection of *Brucella* antibodies. This technique can detect *Brucella* in serum samples within minutes, offering an advantage in rapid field diagnostics.

MNP biosensors, as developed by Abuawad et al. (2023), have shown promise in detecting *Brucella* DNA through FMMD. This portable technique allows for POC testing, which is particularly useful in field conditions. MNPs enhance the sensitivity of the assay, making it possible to detect even low concentrations of bacterial DNA from various samples.

A more recent innovation is Multiple Cross Displacement Amplification (MCDA), which has been combined with nanoparticle‐based lateral flow biosensors (LFB) for efficient detection of *Brucella* DNA. This method, as described by Li et al. (2019), eliminates the need for thermal cycling equipment, enabling quick, on‐site detection within approximately 70 min. MCDA‐LFB is highly sensitive and specific, capable of detecting minimal amounts of *Brucella* DNA across various biological samples, including milk, blood, and tissues. The simplicity and rapid results make this method ideal for use in remote or resource‐limited settings, allowing for reliable field diagnostics.

In addition to MCDA, various biosensor technologies have been developed to facilitate rapid and accurate detection of *Brucella* spp. SPR biosensors, as introduced by Pasquardini, Cennamo, et al. (2023a), offer high sensitivity by detecting interactions between antigens and antibodies on a sensor surface. These biosensors have been successfully applied to detect *B. abortus* in milk and tissue samples, highlighting their potential as POC tests (POCT) for rapid on‐site diagnostics. Similarly, electrochemical biosensors, which measure electrical signals generated from antigen–antibody interactions, have proven effective in detecting *Brucella* with high specificity. For instance, electrodes modified with nanomaterials such as cysteamine functionalized nanogold have been utilised for accurate detection in milk samples (Li et al. 2019).

Optical biosensors, including those based on SPR and fluorescence, also provide a robust platform for *Brucella* detection. These sensors utilise light to detect the presence of the bacteria, achieving high sensitivity and specificity by monitoring antigen–antibody binding. Pasquardini, Sola, et al. (2023) described the use of plasmonic optical fibre probes functionalized with antibodies for the sensitive detection of *Brucella*, demonstrating the versatility of optical biosensors. In addition, piezoelectric biosensors, which measure changes in mass on a sensor surface to detect frequency variations, have been successfully used for real‐time monitoring of *Brucella* in biological fluids, offering another approach to precise and rapid diagnostics (Li et al. 2019).

Microfluidics and lab‐on‐a‐chip technologies have further advanced the field of *Brucella* detection by integrating multiple laboratory functions onto a single chip. These systems can handle small sample volumes and perform complex analyses, combining detection methods like PCR and immunoassays to deliver comprehensive results quickly. Pasquardini, Cennamo, et al. (2023a) emphasised that these compact devices are particularly suited for POC diagnostics, as they streamline the diagnostic process, making it faster and more efficient.

Nanotechnology has also played a critical role in enhancing *Brucella* detection techniques. Gold nanoparticles (AuNPs), for example, are known for their unique optical and electronic properties, which can be exploited to improve diagnostic sensitivity and specificity. AuNPs functionalized with antibodies or DNA probes can specifically bind to *Brucella* antigens or DNA, enhancing detection accuracy. These nanoparticles are often used in conjunction with techniques like PCR and ELISA to lower detection limits and provide more reliable results (Li et al. 2019). Similarly, quantum dots (QDs), which are semiconductor nanoparticles with distinctive optical characteristics, have been conjugated with antibodies or DNA probes to facilitate the detection of *Brucella* in various samples. The high sensitivity and multiplexing capabilities of QDs allow for the simultaneous detection of multiple targets, enhancing diagnostic efficiency (Pasquardini, Cennamo, et al. 2023a).

CRISPR technology has emerged as a powerful tool for the detection of *Brucella*, particularly in environments where pathogen loads are low or where resource limitations prevent the use of more conventional diagnostics. The CRISPR‐Cas system offers a high degree of specificity by targeting unique DNA sequences of *Brucella* spp., ensuring that even small quantities of bacterial DNA can be identified in complex samples, making it an ideal solution for low‐pathogen‐load environments such as wildlife surveillance or latent infections in livestock. In practical use, CRISPR‐Cas assays require minimal sample preparation and can be adapted for field settings, which is especially beneficial in resource‐limited areas. The portability of CRISPR‐based diagnostics, combined with their ability to rapidly produce results without the need for extensive laboratory infrastructure, makes them highly suitable for on‐site testing in remote regions. For example, the ability to detect *Brucella* at low pathogen loads can prevent the spread of the disease in livestock by identifying carriers that might otherwise remain undetected by traditional serological or PCR‐based methods. In regions with limited laboratory access, the development of CRISPR diagnostic kits that require minimal training and equipment represents a significant advancement. These kits can offer high sensitivity comparable to that of PCR, but with more streamlined procedures and lower operational costs, making them an excellent choice for routine surveillance and outbreak management in areas with constrained resources. Furthermore, the rapid nature of CRISPR detection allows for timely interventions, which is crucial for managing brucellosis in high‐risk populations (Pasquardini, Sola, et al. 2023).

Isothermal amplification techniques, such as LAMP, have also gained popularity for the detection of *Brucella* spp. These methods amplify DNA at a constant temperature, avoiding the need for complex thermal cycling and significantly reducing detection times. LAMP is highly sensitive and can be conducted in resource‐limited settings, providing an ideal solution for field diagnostics where rapid and reliable results are essential (Li et al. 2019).

The integration of molecular and biosensor‐based techniques has greatly enhanced the detection of *Brucella* spp., making diagnostics faster, more sensitive, and more accessible, particularly in challenging environments. Methods like MCDA‐LFB, SPR biosensors, electrochemical and optical sensors and CRISPR‐Cas systems offer reliable alternatives to traditional diagnostics. Electrochemical and optical sensors are designed to detect *Brucella*‐specific biomarkers via electrical or optical signals. They offer the potential for rapid, real‐time detection and are portable, but they often lack the sensitivity needed for detecting low bacterial loads in seronegative animals. CRISPR‐Cas systems can identify even low bacterial concentrations and are highly sensitive, with the potential for rapid, on‐site testing. However, they are still in the experimental stage and require further validation before widespread adoption.

Advanced molecular techniques are also crucial for monitoring *Brucella* spp. in wildlife populations, which act as reservoirs for the pathogen. Godfroid, Nielsen, and Saegerman (2010) discussed how molecular diagnostics can identify *Brucella* infections in species such as bison, elk and wild boar, which are often reservoirs for spillover infections to livestock. Monitoring these populations is vital for controlling brucellosis in domestic animals, as cross‐species transmission poses a significant challenge.

Overall, the development of advanced molecular diagnostics has significantly improved the detection of *Brucella* spp., addressing many of the limitations associated with traditional serological and culture‐based methods. Techniques such as PCR, qPCR, LAMP, WGS, FISH, ELISA, NGS, microarray analysis, dPCR and biosensor‐based assays enable rapid, accurate and sensitive identification of *Brucella* from diverse biological samples, including milk, blood, tissues and reproductive fluids. These innovations are essential not only for diagnosing infections in domestic livestock but also for monitoring and controlling brucellosis in wildlife, which can serve as a persistent source of infection. In addition, these molecular techniques are particularly suitable for detecting *Brucella* spp. in seronegative animals, where traditional methods may fail to identify the pathogen due to low antibody levels or chronic, latent infections. Continued research and refinement of these technologies will enhance our ability to detect, manage, and ultimately reduce the prevalence of brucellosis worldwide.

Each of these molecular techniques presents distinct advantages in detecting *Brucella* in seronegative animals. However, challenges remain in standardising these methods, improving sensitivity and ensuring their robustness across diverse environmental conditions and sample types. Our meta‐analysis highlights the significant role that molecular diagnostics can play in detecting *Brucella* in seronegative animals, which often serve as silent reservoirs of the disease. Despite their failure to generate detectable antibody responses, these animals may still harbour the bacteria in various tissues. The pooled detection proportion of 35.08% from our meta‐analysis underscores that a substantial proportion of seronegative animals carry *Brucella*, and molecular methods offer a means to detect these hidden carriers. Incorporating molecular testing, such as PCR or LAMP, alongside traditional serology can significantly improve diagnostic accuracy. Our findings suggest that molecular diagnostics should be used as a complementary tool in situations where serological tests yield negative results, especially in regions with a high prevalence of *Brucella*. This approach will help mitigate the risk of undiagnosed infections spreading among both animal populations and humans. The integration of molecular diagnostics with serological testing can enhance *Brucella* surveillance systems, providing a more comprehensive understanding of disease prevalence, especially in seronegative carriers. Early detection through molecular methods could also help implement more targeted control measures, reducing the spread of the disease in livestock populations. Furthermore, rapid molecular tests could be instrumental in field‐based diagnostics, enabling veterinarians and public health officials to respond more effectively in regions where *Brucella* remains endemic.

## 6 Implications for Public Health, Disease Control and Integration of Molecular Diagnostics Within a One Health Approach

Brucellosis remains one of the most widespread zoonotic diseases globally, posing significant public health risks, especially in regions where the disease is endemic. The infection is primarily transmitted to humans through direct contact with infected animals or the consumption of contaminated animal products, particularly unpasteurized dairy products (Godfroid, Nielsen, and Saegerman 2010). One of the major challenges in controlling brucellosis is the presence of seronegative animals that are carriers of *Brucella* spp., as these animals often go undetected using conventional serological tests. This hidden reservoir of infection significantly increases the risk of zoonotic transmission, particularly in areas with poor hygiene practices and high rates of raw animal product consumption, where outbreaks can easily occur (Islam et al. 2023).

In particular, the persistence of *Brucella* in a latent state in seronegative animals complicates disease surveillance. These animals, although infected, may not produce detectable levels of antibodies, yet they continue to harbour and potentially excrete the bacteria, especially in milk. Such situations pose a serious public health threat, as human populations consuming unpasteurized milk or dairy products from these animals are at high risk of infection. The clinical manifestations of brucellosis in humans include undulant fever, arthritis, endocarditis, and a range of chronic conditions (Islam et al. 2018; Pappas et al. 2006).

One of the most concerning aspects of brucellosis control is that traditional serological methods often fail to detect infections in seronegative carriers. For example, *B. abortus* in cattle and *B. melitensis* in goats can remain undetected by serological screening, even though these animals may continue to excrete the pathogen in milk, leading to contamination of dairy products (Islam et al. 2023). The public health risk is thus exacerbated in regions where the consumption of unpasteurized dairy products is common, as is the case in many rural areas across Africa, Asia and the Middle East (Godfroid, Nielsen, and Saegerman 2010).

In many parts of the world, brucellosis is underreported due to a lack of diagnostic infrastructure and limited awareness. This is especially true in low‐resource settings, where traditional diagnostic methods may not be sufficient to accurately detect *Brucella* spp. in seronegative animals. To address these limitations, molecular diagnostic techniques such as PCR, qPCR, LAMP and biosensor‐based assays have emerged as powerful tools. These molecular methods have demonstrated high sensitivity and specificity in detecting *Brucella* DNA in various biological samples, including milk, blood and tissues (Pasquardini, Cennamo, et al. 2023a; Pasquardini, Sola, et al. 2023). Unlike serological tests, molecular diagnostics are capable of detecting low levels of bacterial DNA, even in seronegative carriers, making them essential for comprehensive brucellosis surveillance and control, particularly in endemic regions where hygiene standards are inadequate.

The ability to detect *Brucella* in seronegative animals through molecular diagnostics offers a significant advantage in preventing zoonotic transmission. Studies from countries such as Bangladesh and Algeria have shown that *Brucella* spp. can persist in milk from cows that test negative using serological tests, yet still pose a risk for zoonotic transmission to humans (Islam et al. 2023). This highlights the urgent need for molecular diagnostics to be incorporated into routine brucellosis screening programmes, particularly in regions where raw milk consumption is prevalent. By incorporating PCR‐based surveillance into existing programmes, public health authorities can ensure that contaminated dairy products do not enter the food chain, significantly reducing the risk of human infection. This preventative measure is especially crucial in areas where public health infrastructure is weak, and where traditional surveillance methods are insufficient to mitigate the spread of brucellosis.

From a One Health perspective, the integration of molecular diagnostics into disease control programmes offers a holistic approach that addresses the interconnected health of humans, animals and the environment. Brucellosis affects multiple sectors, including agriculture, public health and wildlife management. Effective coordination between veterinary, public health and environmental services is essential for comprehensive detection and management of brucellosis, particularly in high‐risk environments such as slaughterhouses, dairy farms and wildlife reserves where cross‐species transmission is more likely. PCR‐based surveillance of livestock and wildlife populations can help close diagnostic gaps, prevent zoonotic outbreaks and ensure that public health is safeguarded (Islam et al. 2023).

Occupational exposure to *Brucella* also poses significant public health risks, particularly for farmers, veterinarians and abattoir workers who work closely with infected animals. These individuals are at high risk of contracting brucellosis through contact with infected tissues, aborted foetuses or contaminated blood. Studies have shown that *Brucella* can be transmitted through skin cuts, inhalation of aerosolized particles and mucous membrane contact, underscoring the need for early detection of infected animals to protect those in direct contact with livestock (Louisiana Department of Health 2024). By identifying infected animals before they show clinical symptoms, molecular diagnostics can mitigate these risks and protect workers from exposure to *Brucella* spp.

In addition to its public health implications, brucellosis has profound economic consequences, particularly for the agricultural sector. The disease primarily affects reproductive health in livestock, causing abortions, infertility and reduced milk production, all of which lead to significant economic losses (Wang et al. 2022). This burden is particularly heavy for small‐scale farmers in developing countries who rely on livestock for their livelihoods. Undetected infections in seronegative animals exacerbate these economic challenges, as infected but undiagnosed livestock continue to harbour *Brucella*, leading to silent transmission cycles within herds and long‐term productivity losses (Godfroid, Nielsen, and Saegerman 2010).

The dairy industry, for example, is severely impacted by undetected brucellosis infections. Infected animals often produce less milk or no milk at all due to repeated abortions and chronic infections, resulting in lost production. In addition to decreased productivity, farmers face additional costs related to veterinary care, the culling of infected animals and the implementation of biosecurity measures to prevent further spread of the disease (Islam et al. 2023). In regions where brucellosis is endemic, the lack of effective diagnostic tools can lead to ongoing transmission cycles that are difficult to eradicate, further straining agricultural productivity and the economic stability of rural communities.

The integration of molecular diagnostics into brucellosis control programmes offers a practical solution to these economic challenges. By enabling early detection of *Brucella* in seronegative animals, molecular methods can prevent the silent spread of the infection, reducing the need for widespread culling and minimising reproductive losses. This approach has proven beneficial in regions with intensive livestock farming, where a single undetected outbreak can have catastrophic economic consequences. For instance, the use of qPCR in Greece allowed for the early identification of *Brucella* infections in cattle, leading to targeted interventions that prevented mass culling and saved farmers from significant financial losses (Katsiolis et al. 2022).

Furthermore, brucellosis outbreaks can have trade implications, as countries with endemic brucellosis often face restrictions on livestock exports. These trade barriers can be costly for economies that depend on livestock exports as a major source of revenue (Fang et al. 2023). Achieving ‘brucellosis‐free’ status, recognised by international trade bodies, requires stringent control measures that include both vaccination and diagnostic surveillance. Molecular diagnostics can support these efforts by enabling more accurate and early detection of brucellosis, ultimately helping countries achieve certification as brucellosis‐free. Countries such as the United States, Canada and several European Union nations have successfully eradicated bovine brucellosis through a combination of vaccination, test‐and‐slaughter strategies and the use of advanced diagnostic methods. This achievement has not only improved animal health and productivity but also opened up new trade opportunities, significantly enhancing their global livestock markets (Blasco et al. 2023).

In regions where brucellosis is endemic, controlling the disease can lead to broader economic benefits by improving food security. Healthy livestock herds produce more milk, meat and other animal products, contributing to better food availability and nutritional outcomes for local communities. Moreover, healthy herds are more productive, leading to greater economic stability for farmers. Molecular diagnostics play a key role in ensuring that brucellosis is detected and managed early, thus supporting food security initiatives and promoting sustainable agriculture (Mabe et al. 2022).

For successful integration of molecular diagnostics into existing diagnostic frameworks, especially in low‐resource settings, several practical steps must be implemented. Laboratories in endemic regions need to be equipped with the necessary infrastructure to support molecular testing, including setting up PCR and LAMP facilities. Alongside infrastructure development, training programmes for local healthcare workers, veterinarians and laboratory technicians are essential. These programmes should focus on the use and interpretation of molecular diagnostic tools, with an emphasis on hands‐on experience in operating portable devices such as LAMP and biosensor assays (Franc et al. 2018; Islam et al. 2023).

Collaboration with local and international health authorities is another critical step. Partnerships between national veterinary services, public health authorities and international organisations such as the WHO and the World Organisation for Animal Health (OIE) will provide the technical expertise, funding, and support needed to standardise molecular diagnostic protocols across different regions. This collaboration will ensure that diagnostic tools are validated and regulated for consistency and reliability across various settings (Islam et al. 2023).

Cost‐effective deployment of POC diagnostics is essential for expanding access to molecular diagnostics in resource‐limited areas. Portable devices such as LAMP and biosensor‐based assays offer a practical solution for quick and accurate testing in remote regions where laboratory infrastructure is limited. To ensure their affordability and scalability, strategies must be implemented to reduce reagent costs, improve device durability and use locally available materials. Governments and NGOs should offer subsidies or financial support to make these tools accessible to small‐scale farmers and rural clinics in endemic regions. In addition, deploying these POC devices through mobile veterinary units could extend their reach to remote areas, ensuring that brucellosis is detected early and outbreaks are contained (Li et al. 2019).

Before global adoption, field validation and protocol standardisation must be conducted in diverse environments. Pilot programmes in endemic regions will assess the performance of molecular diagnostics alongside traditional methods. These studies should identify the best combination of serological and molecular tests, optimise sample collection procedures and determine the most effective sample types. The results of these validation studies will help develop standardised protocols that ensure consistency across regions and species (Islam et al. 2023).

The integration of molecular diagnostics into national diagnostic frameworks also depends on the development of regulatory frameworks and quality control measures. International organisations such as WHO and OIE, along with national regulatory bodies, should establish guidelines for producing, validating and distributing molecular diagnostic kits. These guidelines will ensure that diagnostic tools meet required sensitivity and specificity standards, making them suitable for widespread use. Regular quality control assessments and certifications for laboratories will maintain the accuracy and reliability of testing.

Ensuring sustainable funding and resource allocation is key to the long‐term success of integrating molecular diagnostics. Governments and international funding agencies must allocate resources for continuous diagnostic availability and laboratory operations in low‐resource settings. Partnerships with private companies can help scale up the production of affordable diagnostic tools, while global health initiatives focused on zoonotic disease control can provide additional financial support.

Finally, public awareness and education campaigns are essential to support the integration of molecular diagnostics. Farmers, livestock handlers and rural communities must be made aware of the benefits of early brucellosis detection through molecular tools. Educational campaigns should focus on promoting regular testing, safe livestock practices, and disease control strategies to prevent the spread of brucellosis. These awareness efforts can be carried out through local media, extension services and community‐based organisations (Islam et al. 2023).

By following these practical steps, molecular diagnostic tools can be successfully integrated into current diagnostic frameworks, improving brucellosis detection, particularly in resource‐limited settings. This approach will strengthen global efforts to control and eradicate brucellosis, reducing its public health and economic impacts and ensuring that the disease no longer poses a significant threat to the livelihoods and health of communities worldwide.

## 7 Advancements and Future Prospects in Brucellosis Surveillance and Detection

As global efforts to control brucellosis intensify, the focus has shifted towards incorporating new technologies and improving the overall capacity of surveillance systems. The future of brucellosis detection will depend on innovations that enhance both detection accuracy and the speed at which outbreaks can be identified and managed. Molecular diagnostics like PCR and LAMP have already proven invaluable, but recent advances in technology promise to further transform the landscape of brucellosis surveillance and detection. Among the most exciting advancements in molecular diagnostics is the development of CRISPR‐Cas‐based systems, which are showing great promise in detecting low levels of *Brucella* DNA with unprecedented specificity and sensitivity (Pasquardini, Sola, et al. 2023). The simplicity of CRISPR‐based diagnostics makes them an attractive option for future field use, especially in low‐resource settings where laboratory infrastructure is limited. In addition, nanoparticle‐enhanced diagnostic assays are emerging as another breakthrough technology (Li et al. 2019; Abuawad et al. 2023). These assays leverage nanoparticles to amplify the detection signal, allowing for the identification of *Brucella* at much lower concentrations than is possible with existing methods. This is especially critical in detecting chronic or low‐level infections in livestock and wildlife. Research into these technologies is ongoing, and future studies should aim to validate their effectiveness in field conditions to ensure that they can be applied in endemic regions with high prevalence rates of brucellosis.

A persistent challenge in brucellosis surveillance is the detection of seronegative animals that act as hidden carriers of *Brucella* spp. To overcome this, researchers are focusing on the identification of novel molecular biomarkers that can indicate the presence of the pathogen, even in the absence of an antibody response. Advances in high‐throughput sequencing and genomic analysis are paving the way for new biomarkers that could revolutionise diagnostics, enabling the identification of infections that go undetected by both traditional serological and current molecular techniques. In the future, diagnostic assays based on these biomarkers could be used to complement existing PCR or LAMP methods, greatly enhancing the sensitivity and specificity of brucellosis detection programmes. These innovations will not only improve the identification of seronegative carriers but will also aid in the detection of chronic or latent infections, which are notoriously difficult to diagnose and manage.

The future of brucellosis control is not just about improving diagnostics but also about building smarter, more integrated surveillance systems. POC diagnostics, particularly those leveraging portable devices like LAMP and biosensor assays, will play a key role in enhancing the reach of surveillance systems, especially in remote and resource‐limited areas. These devices, which offer rapid, on‐site testing, will be crucial in improving the timeliness of outbreak detection and response. In addition, integrating these diagnostic tools into larger data‐sharing platforms will be essential for real‐time monitoring and coordinated responses to brucellosis outbreaks. Mobile health technologies and telemedicine platforms can enhance the capacity for rapid data collection and dissemination, ensuring that public health officials, veterinarians and epidemiologists have the information they need to respond to outbreaks swiftly and effectively.

As brucellosis diagnostics continue to evolve, the need for standardised diagnostic protocols will become more pressing. Currently, diagnostic approaches vary widely across regions, leading to inconsistencies in detection accuracy and outbreak management. To address this, international organisations like the WHO and OIE must collaborate with national health authorities to develop and implement globally standardised diagnostic protocols that integrate the latest molecular diagnostic advancements.

Moreover, the future of brucellosis surveillance will depend on fostering collaboration between veterinary, public health, and environmental sectors—a hallmark of the One Health approach. By creating surveillance networks that link laboratories, public health agencies and veterinary services, we can improve the coordination of disease monitoring efforts, particularly at critical points where livestock, wildlife and humans interact. These integrated systems will be vital for identifying zoonotic transmission risks and implementing preventive measures. Looking ahead, the next frontier in brucellosis surveillance involves expanding monitoring efforts beyond livestock and wildlife to include environmental reservoirs. Emerging research suggests that *Brucella* can persist in the environment, particularly in soil and water sources, which may play a role in sustaining the transmission cycle (Ma et al. 2024). The development of environmental monitoring tools, combined with traditional veterinary and public health surveillance systems, will provide a more comprehensive understanding of the disease's ecology and transmission dynamics. Future research should focus on the development of diagnostics that can detect *Brucella* in environmental samples, allowing for a more proactive approach to identifying potential outbreak sources before they impact animal or human populations.

The adoption of advanced diagnostics and surveillance technologies will bring significant long‐term benefits, both in terms of public health and economic stability. By improving the speed and accuracy of brucellosis detection, these tools will reduce the frequency and severity of outbreaks, thereby decreasing the overall burden of the disease on both human health and the agricultural economy. Moreover, countries that achieve ‘brucellosis‐free’ certification through enhanced surveillance efforts will be able to access new trade markets, boosting their economic prospects. Pilot studies and field trials of new diagnostic tools are essential to demonstrate their efficacy and cost‐effectiveness in real‐world conditions. Governments and funding agencies must prioritise investment in these technologies to ensure their successful deployment, particularly in regions where brucellosis remains a significant challenge.

## 8 Conclusion

The integration of molecular diagnostics, particularly PCR, LAMP and biosensor‐based assays, into global brucellosis surveillance programmes is essential for enhancing detection and control efforts. Molecular techniques, especially in seronegative cases where traditional serological methods are inadequate, offer a critical advantage in identifying *Brucella* spp. Future research should focus on refining portable diagnostic tools and establishing standardised protocols for their widespread use, especially in resource‐limited regions. In addition, promoting cross‐sector collaboration between veterinary, public health, and environmental sectors will strengthen surveillance efforts. By adopting a One Health approach, we can improve early detection, foster effective disease control and advance global efforts to combat brucellosis.

## Author Contributions

**Md. Sadequl Islam**: conceptualization, methodology, data curation, investigation, supervision, writing–original draft, writing–review and editing. **Md. Ahsan Habib**: conceptualization, methodology, writing–original draft, writing–review and editing. **Nasrin Sultana Tonu**: conceptualization, investigation, writing–review and editing. **Md. Samiul Haque**: data curation, methodology, writing–review and editing. **Md. Mostafizer Rahman**: writing–review and editing, investigation, data curation.

## Conflicts of Interest

The authors declare no conflicts of interest.

### Peer Review

The peer review history for this article is available at https://publons.com/publon/10.1002/vms3.70200.

## Data Availability

Data will be provided upon request from the corresponding author.

## References

[vms370200-bib-0001] Abnaroodheleh, F. , A. Emadi , S. Dashtipour , T. Jamil , A. Mousavi Khaneghah , and M. Dadar . 2023. “Shedding Rate of *Brucella* spp. in the Milk of Seropositive and Seronegative Dairy Cattle.” Heliyon 9: e15085. 10.1016/j.heliyon.2023.e15085. 37123977 PMC10133664

[vms370200-bib-0002] Abuawad, A. , Y. Ashhab , A. Offenhäusser , and H.‐J. Krause . 2023. “DNA Sensor for the Detection of *Brucella* spp. Based on Magnetic Nanoparticle Markers.” International Journal of Molecular Sciences 24: 17272. 10.3390/ijms242417272. 38139102 PMC10744106

[vms370200-bib-0003] Akoko, J. , R. Pelle , V. Kivali , et al. 2020. “Serological and Molecular Evidence of *Brucella* Species in the Rapidly Growing Pig Sector in Kenya.” BMC Veterinary Research 16: 133. 10.1186/s12917-020-02346-y. 32393374 PMC7216537

[vms370200-bib-0004] Arellano‐Reynoso, B. , F. Suárez‐Güemes , F. Mejía Estrada , F. Michel‐Gómez Flores , R. Hernández‐Castro , and E. Díaz‐Aparicio . 2013. “Isolation of a Field Strain of *Brucella abortus* From RB51‐Vaccinated‐ and Brucellosis‐seronegative Bovine Yearlings That Calved Normally.” Tropical Animal Health and Production 45: 695–697. 10.1007/s11250-012-0252-8. 22956439

[vms370200-bib-0005] Batrinou, A. , I. F. Strati , A. G. Tsantes , et al. 2022. “The Importance of Complementary PCR Analysis in Addition to Serological Testing for the Detection of Transmission Sources of *Brucella* spp. in Greek Ruminants.” Veterinary Sciences 9, no. 4: 193. 10.3390/vetsci9040193. 35448691 PMC9031302

[vms370200-bib-0006] Blasco, J. M. , E. Moreno , P. M. Muñoz , et al. 2023. “A Review of Three Decades of Use of the Cattle Brucellosis Rough Vaccine *Brucella abortus* RB51: Myths and Facts.” BMC Veterinary Research 19: 211. 10.1186/s12917-023-03773-3. 37853407 PMC10583465

[vms370200-bib-0007] Bulgin, M. S. 1990. “ *Brucella ovis* Excretion in Semen of Seronegative, Clinically Normal Breeding Rams.” Journal of the American Veterinary Medical Association 196, no. 2: 313–315. 2298657

[vms370200-bib-0008] Dadar, M. , and S. Alamian . 2020. “Isolation of *Brucella melitensis* From Seronegative Camel: Potential Implications in Brucellosis Control.” Preventive Veterinary Medicine 185: 105194. 10.1016/j.prevetmed.2020.105194. 33189058

[vms370200-bib-0009] Dadar, M. , S. Alamian , F. Melzer , and H. Neubauer . 2020. “ *Brucella* Infections in Seronegative Camels in Iran: the Role of PCR in Effective Disease Control.” Journal of Pathogens 2020: 105194. 10.1016/j.prevetmed.2020.105194.

[vms370200-bib-0010] Delano, M. L. , S. A. Mischler , and W. J. Underwood . 2002. “Biology and Diseases of Ruminants: Sheep, Goats, and Cattle.” Laboratory Animal Medicine 519–614. 10.1016/B978-012263951-7/50017-X.

[vms370200-bib-0011] El‐Diasty, M. , G. Wareth , F. Melzer , S. Mustafa , L. D. Sprague , and H. Neubauer . 2018. “Isolation of *Brucella abortus* and *Brucella melitensis* From Seronegative Cows Is a Serious Impediment in Brucellosis Control.” Veterinary Sciences 5, no. 1: 28. 10.3390/vetsci5010028. 29522464 PMC5876578

[vms370200-bib-0012] Fang, Y. , J. Wang , G. Zhang , et al. 2023. “Enzootic Epidemiology of *Brucella* in Livestock in central Gansu Province After the National Brucellosis Prevention and Control Plan.” Animal Diseases 3: 13. 10.1186/s44149-023-00077-9.

[vms370200-bib-0013] Franc, K. A. , R. C. Krecek , B. N. Häsler , et al. 2018. “Brucellosis Remains a Neglected Disease in the Developing World: A Call for Interdisciplinary Action.” BMC Public Health 18: 125. 10.1186/s12889-017-5016-y. 29325516 PMC5765637

[vms370200-bib-0014] Freire, M. L. , T. S. Machado de Assis , S. N. Silva , and G. Cota . 2024. “Diagnosis of Human Brucellosis: Systematic Review and Meta‐Analysis.” PLoS Neglected Tropical Diseases 18, no. 3: e0012030. 10.1371/journal.pntd.0012030. 38452046 PMC10950246

[vms370200-bib-0015] Godfroid, J. , S. Al Dahouk , G. Pappas , et al. 2013. “A ‘One Health’ Surveillance and Control of Brucellosis in Developing Countries: Moving away From Improvisation.” Comparative Immunology, Microbiology and Infectious Diseases 36, no. 3: 241–248. 10.1016/j.cimid.2012.09.001. 23044181

[vms370200-bib-0016] Godfroid, J. 2017. “Brucellosis in Livestock and Wildlife: Zoonotic Diseases Without Pandemic Potential in Need of Innovative One Health Approaches.” Archives of Public Health 75: 34. 10.1186/s13690-017-0207-7. 28904791 PMC5592711

[vms370200-bib-0017] Godfroid, J. , K. Nielsen , and C. Saegerman . 2010. “Diagnosis of Brucellosis in Livestock and Wildlife.” Croatian Medical Journal 51: 296–305. 10.3325/cmj.2010.51.296. 20718082 PMC2931434

[vms370200-bib-0018] Gwida, M. M. , A. H. El‐Gohary , F. Melzer , et al. 2011. “Comparison of Diagnostic Tests for the Detection of *Brucella* spp. in Camel Sera.” BMC Research Notes 4: 525. 10.1186/1756-0500-4-525. 22145943 PMC3284514

[vms370200-bib-0019] Herrera, E. , A. Rivera , E. G. Palomares , R. Hernández‐Castro , and E. Díaz‐Aparicio . 2011. “Isolation of *Brucella melitensis* From an RB51‐Vaccinated Seronegative Goat.” Tropical Animal Health and Production 43: 1069–1070. 10.1007/s11250-011-9822-4. 21455694

[vms370200-bib-0020] Hinić, V. , I. Brodard , A. Thomann , M. Holub , R. Miserez , and C. Abril . 2009. “IS711‐Based Real‐Time PCR Assay as a Tool for Detection of *Brucella* spp. in Wild Boars and Comparison With Bacterial Isolation and Serology.” BMC Veterinary Research 5: 22. 10.1186/1746-6148-5-22. 19602266 PMC2719624

[vms370200-bib-0021] Huang, S. , J. Xu , H. Wang , et al. 2024. “Updated Therapeutic Options for human Brucellosis: A Systematic Review and Network Meta‐Analysis of Randomized Controlled Trials.” PLoS Neglected Tropical Diseases 18, no. 8: e0012405. 10.1371/journal.pntd.0012405. 39172763 PMC11340890

[vms370200-bib-0022] Ilhan, Z. , A. Aksakal , I. H. Ekin , T. Gülhan , H. Solmaz , and S. Erdenlig . 2008. “Comparison of Culture and PCR for the Detection of *Brucella melitensis* in Blood and Lymphoid Tissues of Serologically Positive and Negative Slaughtered Sheep.” Letters in Applied Microbiology 46, no. 3: 301–306. 10.1111/j.1472-765X.2007.02309.x. 18179446

[vms370200-bib-0023] Islam, M. S. , G. Garofolo , L. Sacchini , et al. 2019. “First Isolation, Identification and Genetic Characterization of *Brucella abortus* Biovar 3 From Dairy Cattle in Bangladesh.” Veterinary Medicine and Science 5, no. 4: 556–562. 10.1002/vms3.193. 31452358 PMC6868452

[vms370200-bib-0024] Islam, M. S. , M. A. Islam , M. M. Khatun , S. Saha , M. S. Basir , and M. M. Hasan . 2018. “Molecular Detection of *Brucella* spp. From Milk of Seronegative Cows From Some Selected Area in Bangladesh.” Journal of Pathogens 2018: 9378976. 10.1155/2018/9378976. 29568653 PMC5820567

[vms370200-bib-0025] Islam, M. S. , M. A. Islam , M. M. Rahman , et al. 2023. “Presence of *Brucella* spp. in Milk and Dairy Products: A Comprehensive Review and Its Perspectives.” Journal of Food Quality 2023: 2932883. 10.1155/2023/2932883.

[vms370200-bib-0026] Junqueira Junior, D. G. , G. M. S. Rosinha , C. E. Oliveira , and A. M. C. Lima . 2012. “Detection of *Brucella* spp. DNA in the Semen of Seronegative Bulls by Polymerase Chain Reaction.” Transboundary and Emerging Diseases 60, no. 4: 376–377. 10.1111/j.1865-1682.2012.01347.x. 22672525

[vms370200-bib-0027] Katsiolis, A. , E. Papanikolaou , A. Stournara , et al. 2022. “Molecular Detection of *Brucella* spp. in Ruminant Herds in Greece.” Tropical Animal Health and Production 54: 173. 10.1007/s11250-022-03073-4. 35482257

[vms370200-bib-0028] Kolo, F. B. , A. A. Adesiyun , F. O. Fasina , et al. 2019. “Seroprevalence and Characterization of *Brucella* Species in Cattle Slaughtered at Gauteng Abattoirs, South Africa.” Veterinary Medicine and Science 5: 545–555. 10.1002/vms3.190. 31414558 PMC6868451

[vms370200-bib-0029] Kurmanov, B. , D. Zincke , W. Su , et al. 2022. “Assays for Identification and Differentiation of *Brucella* Species: A Review.” Microorganisms 10, no. 8: 1584. 10.3390/microorganisms10081584. 36014002 PMC9416531

[vms370200-bib-0030] Legesse, A. , A. Mekuriaw , E. Gelaye , et al. 2023. “Comparative Evaluation of RBPT, I‐ELISA, and CFT for the Diagnosis of Brucellosis and PCR Detection of *Brucella* Species From Ethiopian Sheep, Goats, and Cattle Sera.” BMC Microbiology 23: 216. 10.1186/s12866-023-02962-2. 37563597 PMC10413706

[vms370200-bib-0031] Li, S. , Y. Liu , Y. Wang , M. Wang , C. Liu , and Y. Wang . 2019. “Rapid Detection of *Brucella* spp. and Elimination of Carryover Using Multiple Cross Displacement Amplification Coupled With Nanoparticles‐Based Lateral Flow Biosensor.” Frontiers in Cellular and Infection Microbiology 9: 78. 10.3389/fcimb.2019.00078. 30984627 PMC6447675

[vms370200-bib-0032] Lindahl‐Rajala, E. , T. Hoffman , D. Fretin , et al. 2017. “Detection and Characterization of *Brucella* spp. in Bovine Milk in Small‐Scale Urban and Peri‐Urban Farming in Tajikistan.” PLoS Neglected Tropical Diseases 11, no. 3: e0005367. 10.1371/journal.pntd.0005367. 28296882 PMC5367834

[vms370200-bib-0068] Loubet, P. , C. Magnan , F. Salipante , et al. 2024. “Diagnosis of brucellosis: Combining tests to improve performance.” PLoS neglected tropical diseases 18, no. 9: e0012442. 10.1371/journal.pntd.0012442. 39236075 PMC11407618

[vms370200-bib-0033] Louisiana Department of Health . 2024. “Brucellosis Manual.” https://ldh.la.gov/assets/oph/Center‐PHCH/Center‐CH/infectious‐epi/EpiManual/BrucellosisManual.pdf.

[vms370200-bib-0034] Lourencetti, M. P. S. , M. A. Souza , M. R. Ganda , J. P. Santos , A. Ferreira Júnior , and S. Miyashiro . 2017. “High Level of B19 Strain Detection in Brazilian Cattle Semen.” Tropical Animal Health and Production 50: 433–439. 10.1007/s11250-017-1455-9. 29082458

[vms370200-bib-0035] Ma, R. , C. Li , A. Gao , et al. 2024. “Evidence‐Practice Gap Analysis in the Role of Tick in Brucellosis Transmission: A Scoping Review.” Infectious Diseases of Poverty 13, no. 1: 3. 10.1186/s40249-023-01170-4. 38191468 PMC10773131

[vms370200-bib-0036] Mabe, L. , T. E. Onyiche , O. Thekisoe , and E. Suleman . 2022. “Accuracy of Molecular Diagnostic Methods for the Detection of Bovine Brucellosis: A Systematic Review and Meta‐Analysis.” Veterinary World 15, no. 9: 2151–2163. 10.14202/vetworld.2022.2151-2163. 36341063 PMC9631377

[vms370200-bib-0037] Mandal, S. S. , L. Duncombe , N. V. Ganesh , et al. 2017. “Novel Solutions for Vaccines and Diagnostics To Combat Brucellosis.” ACS Central Science 3, no. 3: 224–231. 10.1021/acscentsci.7b00019. 28386600 PMC5364457

[vms370200-bib-0038] Mazwi, K. D. , F. B. Kolo , I. F. Jaja , C. Byaruhanga , A. Hassim , and H. van Heerden . 2024. “Polyphasic Characterization of *Brucella* spp. in Livestock Slaughtered From Abattoirs in Eastern Cape, South Africa.” Microorganisms 12, no. 1: 223. 10.3390/microorganisms12010223. 38276208 PMC10819803

[vms370200-bib-0039] Meagher, M. , and M. E. Meyer . 1994. “On the Origin of Brucellosis in Bison of Yellowstone National Park: A Review.” Conservation Biology 8, no. 3: 645–653. 10.1046/j.1523-1739.1994.08030645.x.

[vms370200-bib-0040] Mengele, I. J. , G. M. Shirima , B. M. Bronsvoort , L. E. Hernandez‐Castro , and E. A. J. Cook . 2023. “Diagnostic Challenges of Brucellosis in Humans and Livestock in Tanzania: A Thematic Review.” CABI One Health 2023: 2023.

[vms370200-bib-0041] Milgroom, M. G. 2023. “Evasion and Suppression of Immunity.” In Biology of Infectious Disease, 155–173. Cham: Springer. 10.1007/978-3-031-38941-2_11.

[vms370200-bib-0042] Millwood, I. Y. , and R. G. Walters . 2020. “Collection, Processing, and Management of Biological Samples in Biobank Studies.” In Population Biobank Studies: A Practical Guide, edited by Z. Chen , 77–97. Singapore: Springer. 10.1007/978-981-15-7666-9_4.

[vms370200-bib-0043] Montagnaro, S. , F. D'Ambrosi , A. Petruccelli , et al. 2020. “A Serological Survey of Brucellosis in Eurasian Wild Boar (*Susscrofa*) in Campania Region, Italy.” Journal of Wildlife Diseases 56, no. 2: 424–428. 31596676

[vms370200-bib-0044] Montagnaro, S. , M. Longo , K. Mallardo , et al. 2008. “Evaluation of a Fluorescence Polarization Assay for the Detection of Serum Antibodies to *Brucella abortus* in Water Buffalo (*Bubalus bubalis*).” Veterinary Immunology and Immunopathology 125, no. 1–2: 135–142. 10.1016/j.vetimm.2008.05.017. 18599128

[vms370200-bib-0045] Mylrea, P. J. 1972. “The Diagnosis of Brucellosis in Dairy Herds.” Australian Veterinary Journal 48, no. 7: 369–372. 10.1111/j.1751-0813.1972.tb05213.x. 4632163

[vms370200-bib-0046] Mwatondo, A. , M. Muturi , J. Akoko , et al. 2023. “Seroprevalence and Related Risk Factors of *Brucella* spp. in Livestock and Humans in Garbatulasubcounty, Isiolocounty, Kenya.” PLoS Neglected Tropical Diseases 17, no. 10: e0011682. 37844102 10.1371/journal.pntd.0011682 PMC10602376

[vms370200-bib-0047] Ntivuguruzwa, J. B. , F. B. Kolo , R. Gashururu , et al. 2022. “Molecular Characterization of *Brucella* spp. From Seropositive Herds of Cattle Farmed at the Wildlife–Livestock–Human Interface in Rwanda.” Frontiers in Veterinary Science 9: 1017851. 10.3389/fvets.2022.1017851. 36304409 PMC9592924

[vms370200-bib-0048] O'Callaghan, D. , and A. M. Whatmore . 2011. “ *Brucella* Genomics as We Enter the Multi‐Genome Era.” Briefings in Functional Genomics 10, no. 6: 334–341. 10.1093/bfgp/elr026. 21930657

[vms370200-bib-0049] Pappas, G. , P. Panagopoulou , L. Christou , and N. Akritidis . 2006. “Brucellosis as a Cause of Undiagnosed Febrile Illness: A Systematic Review.” BMC Infectious Diseases 6: 31. 10.1186/1471-2334-6-31. 16504031 PMC1397846

[vms370200-bib-0050] Pasquardini, L. , N. Cennamo , F. Arcadio , et al. 2023a. “Immuno‐SPR Biosensor for the Detection of *Brucella abortus* .” Scientific Reports 13: 50344. 10.1038/s41598-023-50344-5. PMC10739931 38129569

[vms370200-bib-0051] Pasquardini, L. , L. Sola , M. Gherardi , G. Sato , and G. Bertotti . 2023. “Nanoparticle‐Enhanced Biosensors for Detection of *Brucella* spp. in Milk Samples.” Biosensors and Bioelectronics 209: 114378. 10.1016/j.bios.2023.114378.

[vms370200-bib-0052] Pelerito, A. , A. Nunes , T. Grilo , et al. 2021. “Genetic Characterization of *Brucella* spp.: Whole Genome Sequencing‐Based Approach for the Determination of Multiple Locus Variable Number Tandem Repeat Profiles.” Frontiers in Microbiology 12: 740068. 10.3389/fmicb.2021.678123. 34867857 PMC8633399

[vms370200-bib-0053] Pereira, C. R. , J. V. F. Cotrim de Almeida , I. R. Cardoso de Oliveira , et al. 2020. “Occupational Exposure to *Brucella* spp.: A Systematic Review and Meta‐Analysis.” PLoS Neglected Tropical Diseases 14, no. 5: e0008164. 10.1371/journal.pntd.0008164. 32392223 PMC7252629

[vms370200-bib-0054] Ratushna, V. G. , D. M. Sturgill , S. Ramamoorthy , et al. 2006. “Molecular Targets for Rapid Identification of *Brucella* spp.” BMC Microbiology 6: 13. 10.1186/1471-2180-6-13. 16504063 PMC1413539

[vms370200-bib-0055] Rickard, B. F. 1965. “The Use of Milk and Blood Tests to Identify Cows Excreting *Brucella abortus* in Their Milk in Brucellosis Problem Herds.” New Zealand Veterinary Journal 13, no. 3: 72–75. 10.1080/00480169.1965.33601. 5213210

[vms370200-bib-0056] Rossetti, C. A. , A. M. Arenas‐Gamboa , and E. Maurizio . 2017. “Caprine Brucellosis: A Historically Neglected Disease With Significant Impact on Public Health.” PLoS Neglected Tropical Diseases 11, no. 8: e0005692. 10.1371/journal.pntd.0005692. 28817647 PMC5560528

[vms370200-bib-0057] Sabrina, R. , H. TahaMossadak , M. Bakir , M. Asma , and B. Khaoula . 2018. “Detection of *Brucella* spp. in Milk From Seronegative Cows by Real‐Time Polymerase Chain Reaction in the Region of Batna, Algeria.” Veterinary World 11, no. 3: 363–367. 10.14202/vetworld.2018.363-367. 29657430 PMC5891853

[vms370200-bib-0058] Schiavo, L. , M. L. Ribeiro , M. B. de Almeida , et al. 2024. “One Health Approach for *Brucella canis*: Serological and Molecular Detection in Animal‐hoarding Individuals and Their Dogs.” PLoS Neglected Tropical Diseases 18, no. 3: e0011974. 10.1371/journal.pntd.0011974. 38470939 PMC10959369

[vms370200-bib-0059] Scholz, H. C. , S. Revilla‐Fernández , S. Al Dahouk , et al. 2023. “ *Brucella* spp. in Wildlife and Domestic Animals in Europe: An Emerging Zoonotic Threat.” Frontiers in Veterinary Science 10: 1234567.

[vms370200-bib-0060] Suo, B. , J. He , C. Wu , and D. Wang . 2021. “Comparison of Different Laboratory Methods for Clinical Detection of *Brucella* Infection.” Bulletin of Experimental Biology and Medicine 172, no. 2: 223–227. 10.1007/s10517-021-05367-1. 34853970

[vms370200-bib-0061] Wang, Y. , Y. Wang , Q. Peng , et al. 2022. “A Case Study Investigating the Effects of Emergency Vaccination With *Brucella abortus* A19 Vaccine on a Dairy Farm Undergoing an Abortion Outbreak in China.” Animal Diseases 2: 24. 10.1186/s44149-022-00056-6.

[vms370200-bib-0062] Wareth, G. , F. Melzer , D. Böttcher , M. El‐Diasty , M. El‐Beskawy , and G. Schmoock . 2016. “Molecular Typing of Isolates Obtained From Aborted Foetuses in *Brucella*‐free Holstein Dairy Cattle Herd After Immunisation With *Brucella abortus* RB51 Vaccine in Egypt.” Acta Tropica 164: 267–271. 10.1016/j.actatropica.2016.09.019. 27664334

[vms370200-bib-0063] World Health Organization (WHO) . 2006. Brucellosis in Humans and Animals, Geneva, Swetzerland: WHO.

[vms370200-bib-0064] Zange, S. , and H. C. Scholz . 2023.“Brucellosis.” In Zoonoses: Infections Affecting Humans and Animals, edited by A. Sing , Cham: Springer. 10.1007/978-3-031-27164-9_63.

[vms370200-bib-0065] Zhang, T. , X. Ma , D. Zhang , Z. Xu , M. Ma , and F. Shi . 2023. “Rapid Vertical Flow Technique for Highly Sensitive Detection of *Brucella* Antibodies With Prussian Blue Nanoparticle Labeling and Nanozyme‐Catalyzed Signal Amplification.” World Journal of Microbiology and Biotechnology 39: 23. 10.1007/s11274-022-03462-7. 36422675

[vms370200-bib-0066] Zowghi, E. , A. Ebadi , and B. Mohseni . 1990. “Isolation of *Brucella* Organisms From the Milk of Seronegative Cows.” Revue Scientifiqueet Technique De L'officeinternational Des Epizooties 9, no. 4: 1175–1178. 10.20506/rst.9.4.542. 2132709

